# The emerging role of RNA modifications in the regulation of mRNA stability

**DOI:** 10.1038/s12276-020-0407-z

**Published:** 2020-03-24

**Authors:** Sung Ho Boo, Yoon Ki Kim

**Affiliations:** 1 Creative Research Initiatives Center for Molecular Biology of Translation, Korea University, Seoul, 02841 Republic of Korea; 2 Division of Life Sciences, Korea University, Seoul, 02841 Republic of Korea

**Keywords:** RNA modification, RNA decay

## Abstract

Many studies have highlighted the importance of the tight regulation of mRNA stability in the control of gene expression. mRNA stability largely depends on the mRNA nucleotide sequence, which affects the secondary and tertiary structures of the mRNAs, and the accessibility of various RNA-binding proteins to the mRNAs. Recent advances in high-throughput RNA-sequencing techniques have resulted in the elucidation of the important roles played by mRNA modifications and mRNA nucleotide sequences in regulating mRNA stability. To date, hundreds of different RNA modifications have been characterized. Among them, several RNA modifications, including *N*^*6*^-methyladenosine (m^6^A), *N*^*6*^,2′-*O*-dimethyladenosine (m^6^Am), 8-oxo-7,8-dihydroguanosine (8-oxoG), pseudouridine (Ψ), 5-methylcytidine (m^5^C), and *N*^*4*^-acetylcytidine (ac^4^C), have been shown to regulate mRNA stability, consequently affecting diverse cellular and biological processes. In this review, we discuss our current understanding of the molecular mechanisms underlying the regulation of mammalian mRNA stability by various RNA modifications.

## Gene activity: the significance of RNA modification

Messenger RNA molecules play their key role in directing the manufacture of proteins via translating the genetic information in DNA. Sung Ho Boo and Yoon Ki Kim at Korea University in Seoul review current understanding of the significance of modifications to messenger RNA with respect to RNA stability in mammalian cells. Recent advances in RNA sequencing technology have revealed hundreds of different modifications which, by influencing RNA stability, can control gene expression. The modifications largely involve small chemical groups added to any of the four nucleotide groups that make up an RNA molecule. This can affect all aspects of mature RNA biogenesis, including transcription, pre-mRNA splicing, RNA export form the nucleus to the cytoplasm, translation, and stability.

## Introduction

Many recent studies have demonstrated that RNA undergoes various modifications in a manner similar to DNA. These RNA modifications play a role in many cellular and biological processes, thereby opening up an emerging research field known as epitranscriptomics^1–7^. According to the MODOMICS database, ~170 different RNA modifications have been identified in coding and noncoding RNAs^5,8,9^. In certain types of modifications, the specific nucleotide sequences and positions targeted for RNA modification have been well characterized due to recent advances in specialized high-throughput RNA-sequencing technologies^10^.

**Fig. 1:**
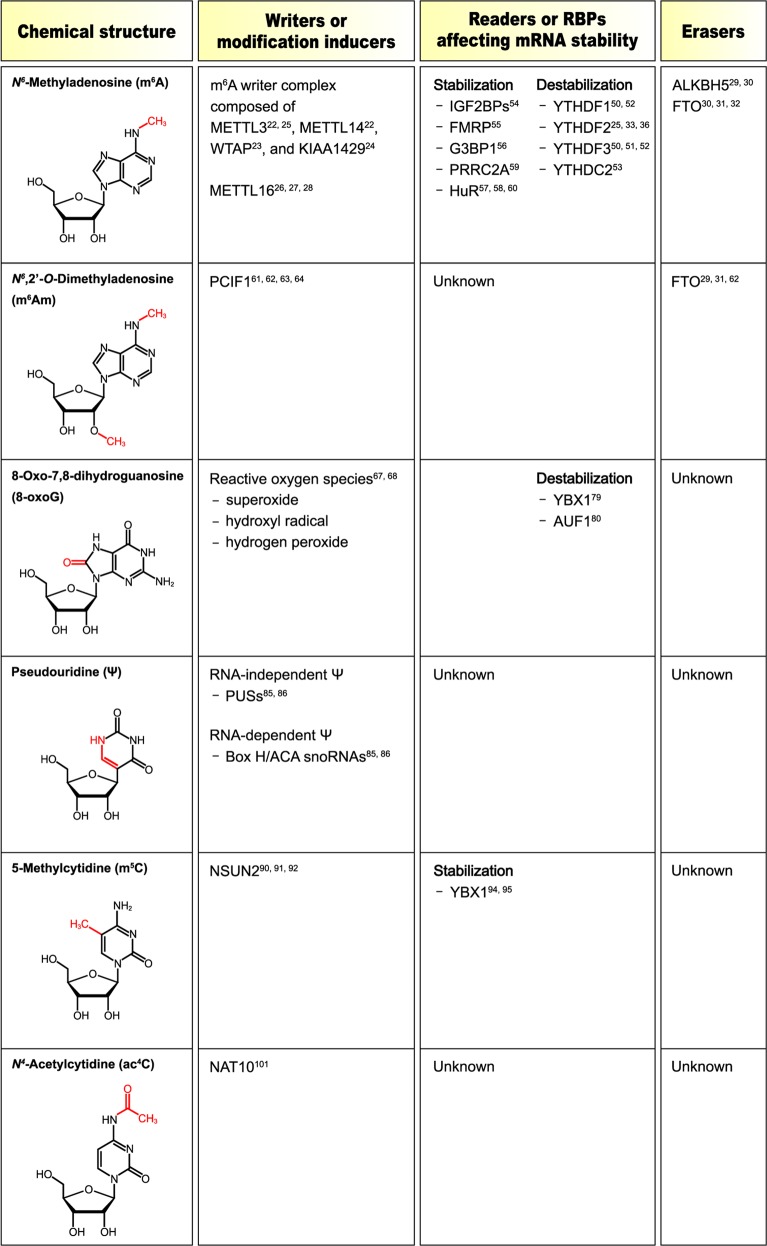
Chemical structures of RNA modifications affecting mRNA stability. The chemical structures of the six RNA modifications discussed in this review are shown. The modified chemical groups are depicted in red. The known writers (or modification inducers), readers (or RBPs) involved in mRNA stability, and erasers for each RNA modification are also summarized.

Generally, the fate of a modified transcript is determined by the coordinated actions of the following three effector proteins (Fig. 1)^1,3–7^: (i) writer proteins (RNA-modifying enzymes), which transfer a specific chemical group to a target position on an RNA molecule; (ii) RNA-binding proteins (RBPs), which specifically recognize the modified nucleotides (reader proteins); and (iii) eraser proteins, which remove specific chemical groups from the modified nucleotides, converting them back into unmodified nucleotides. In certain cases, endogenous or exogenous chemical damage can also generate RNA modifications without the involvement of writer proteins^11,12^. In addition, some modifications are reversible, while others are irreversible.

These RNA modifications can affect a variety of molecular processes, such as transcription, pre-mRNA splicing, RNA export, mRNA translation, and RNA degradation^1,3–7^. All of these molecular events contribute to shaping the cellular transcriptome and proteome^1–7,13^. In particular, recent reports have posited that the regulation of mRNA stability through RNA modification is a crucial step for the tight regulation of gene expression^1–7,13^. Therefore, in this review, we aim to highlight recent progress made in our understanding of the molecular mechanisms underlying the regulation of mRNA stability through various mRNA modifications, including *N*^6^-methyladenosine (m^6^A), *N*^*6*^,2′-*O*-dimethyladenosine (m^6^Am), 8-oxo-7,8-dihydroguanosine (8-oxoG), pseudouridine (Ψ), 5-methylcytidine (m^5^C), and *N*^*4*^-acetylcytidine (ac^4^C).

### *N*^*6*^-methyladenosine

m^6^A is the most abundant internal mRNA modification, and it affects various cellular and physiological processes, such as maternal-to-zygotic transition (MZT), cortical neurogenesis, and the regulation of cancer stem cells in acute myeloid leukemia^7,14–19^. A transcriptome-wide analysis for identifying the consensus sequence motifs for the m^6^A modification in the human transcriptome revealed that m^6^A sites (Gm^6^AC or Am^6^AC) are found in noncoding RNAs and mRNAs, with a greater number within long exons and adjacent to stop codons^20,21^.

The m^6^A modification is cotranscriptionally generated in nascent transcripts by a methyltransferase complex comprising methyltransferase like 3 (METTL3), METTL14, WTAP, and KIAA1429 (Fig. 1)^7,17–19,22–25^. The methyltransferase complex transfers a methyl group to the N-6 position of the adenosine base. The second m^6^A writer protein, METTL16 also contributes to m^6^A modification of both coding and noncoding RNAs^26–28^. However, only a handful of mRNAs, such as *MAT2A* mRNA, have been identified as substrates of METTL16. m^6^A is reversibly converted into adenosine by m^6^A erasers (demethylases), which remove the methyl group from m^6^A. The α-ketoglutarate-dependent dioxygenase alkB homolog 5 protein (ALKBH5) is known to be the primary and specific m^6^A demethylase^29,30^. In addition, fat mass and obesity-associated protein (FTO) demethylase is known to have a weak preference for m^6^A^30–32^.

**Fig. 2:**
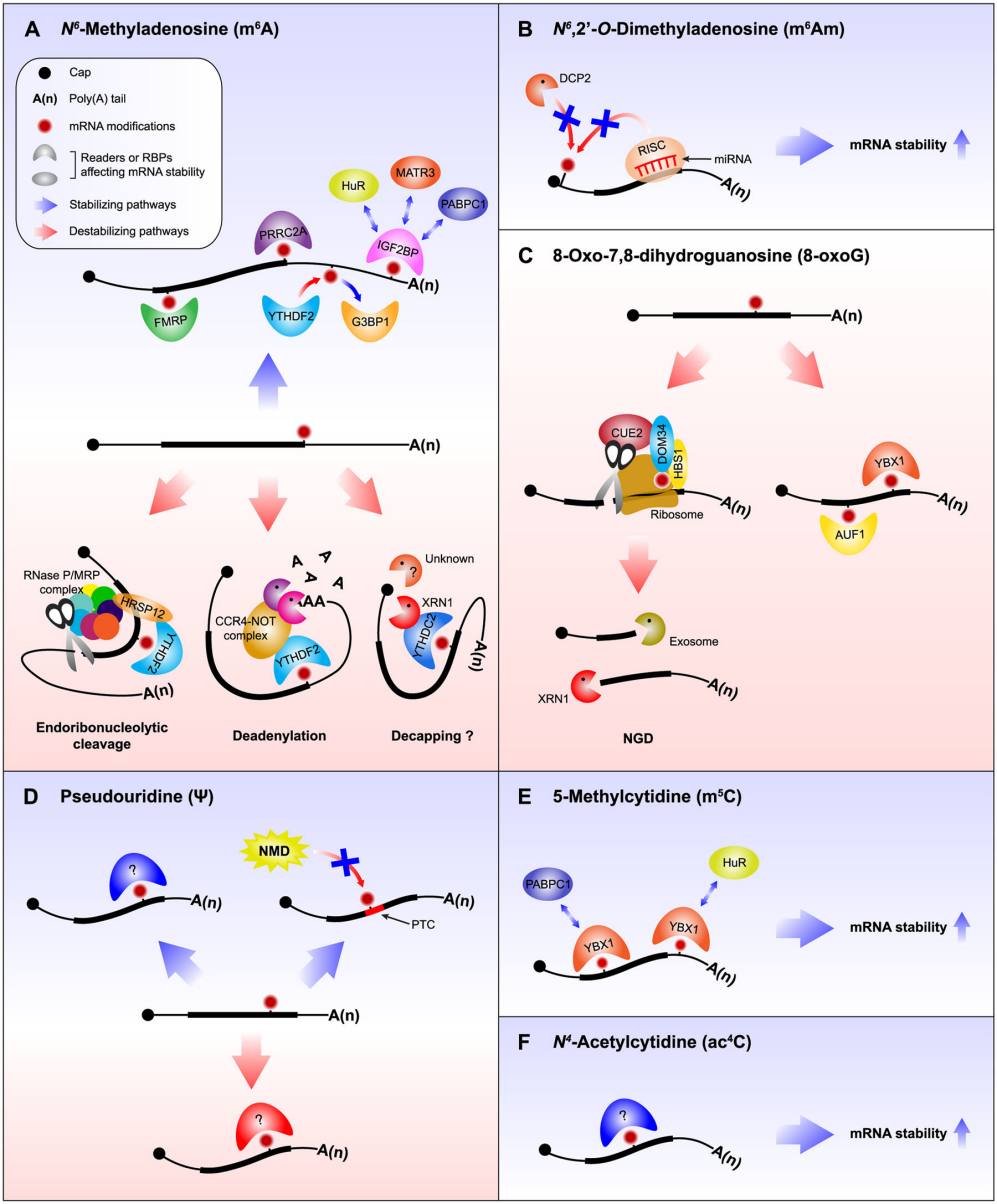
Molecular mechanisms underlying the regulation of mRNA stability through diverse RNA modifications. **a** *N*^*6*^-methyladenosine (m^6^A): in general, YTH domain-containing proteins destabilize m^6^A-containing mRNAs. When m^6^A is recognized by YTHDF2, the degradation of m^6^A-containing mRNAs is initiated by deadenylation through the CCR4–NOT complex. If the YTHDF2-bound m^6^A-containing mRNA harbors an HRSP12-binding site, the degradation of the mRNA is preferentially initiated through an endoribonucleolytic cleavage reaction mediated by the RNase P/MRP complex. YTHDC2 binds to m^6^A and recruits XRN1, thereby triggering 5′–3′ exoribonucleolytic cleavage. In contrast, m^6^A-containing mRNA can be stabilized by other m^6^A reader proteins or RBPs, including IGF2BP, FMRP, G3BP1, PRRC2A, and HuR. **b** *N*^*6*^,2′-*O*-dimethyladenosine (m^6^Am): the presence of m^6^Am at the 5′-end of mRNA blocks its accessibility to DCP2, thus stabilizing the mRNA. m^6^Am also enables mRNA to become more resistant to microRNA-mediated mRNA degradation. **c** 8-Oxo-7,8-dihydroguanosine (8-oxoG): the presence of 8-oxoG in mRNA causes ribosome stalling, thereby triggering NGD. Alternatively, 8-oxoG-containing mRNAs are degraded through 8-oxoG reader proteins, such as YBX1 and AUF1. **d** Pseudouridine (Ψ): Ψ can stabilize or destabilize mRNA. In particular, Ψ on PTCs results in the inhibition of NMD. As a consequence, the mRNA is stabilized. **e** 5-Methylcytidine (m^5^C): YBX1 specifically recognizes m^5^C on mRNA and recruits either PABPC1 or HuR, thereby stabilizing the mRNA. **f** *N*^4^-acetylcytidine (ac^4^C). The presence of ac^4^C stabilizes mRNA by unknown mechanisms.

The m^6^A modification plays a regulatory role in diverse molecular processes, such as transcription, pre-mRNA splicing, mRNA export, mRNA stability, and translation^7,17–19^. The molecular events that occur through the m^6^A modification are guided by various m^6^A-recognizing reader proteins, such as YT521-B homology (YTH) domain-containing proteins. The YTHDF2 protein is the most representative m^6^A reader protein involved in the decay of m^6^A-containing RNA (Fig. 2)^25^. YTHDF2 contains a P/Q/N-rich unstructured region in its N-terminal half, which is critical for YTHDF2 interactions with other cellular factors, and an RNA-binding domain in the C-terminal half, which is crucial for binding m^6^A-containing transcripts^25,33–35^. When an m^6^A-containing mRNA is recognized by YTHDF2, rapid degradation of mRNA is initiated in one of two distinct pathways: the deadenylation pathway or the endoribonucleolytic pathway^19,33,36^. Rapid deadenylation of m^6^A-containing mRNA is accelerated by the recruitment of a deadenylase complex (CCR4–NOT complex)^37,38^ to the m^6^A-containing mRNA via a direct interaction with the N-terminus of YTHDF2 and the SH domain of CNOT1, a component of the CCR4–NOT complex^33^. The resulting deadenylated m^6^A-containing mRNA would be more vulnerable to 3′–5′ exoribonucleolytic cleavage by the exosome complex or DIS3-like enzymes^39–43^.

As an alternative to deadenylation followed by 3′–5′ exoribonucleolytic cleavage, endoribonucleolytic cleavage of m^6^A-containing mRNAs can be initiated by the interplay among YTHDF2, heat-responsive protein 12 (HRSP12, also known as reactive intermediate imine deaminase A homolog), and an endoribonuclease RNase P/MRP complex (Fig. 2)^36^. HRSP12 has been identified as a cellular factor involved in glucocorticoid receptor-mediated mRNA decay^43–46^. The RNase P/MRP complex has been characterized as an endoribonuclease that cleaves long noncoding RNAs and mRNAs, as well as precursor forms of 5.8 S rRNA and tRNA^47–49^. When YTHDF2 binds to an m^6^A-containing mRNA, it recruits HRSP12, which functions as an adaptor protein that links YTHDF2 and POP1, a component of the RNase P/MRP complex^36^. Results from transcriptome analyses have shown that HRSP12 preferentially binds to the GGUUC motif, typically located upstream of YTHDF2-binding sites^36^. Intriguingly, HRSP12 and YTHDF2 bind to mRNA in a cooperative manner^36^. This cooperative interaction facilitates the efficient recruitment of the RNase P/MRP complex to the m^6^A-containing mRNA. Consequently, the recruited RNase P/MRP complex triggers endoribonucleolytic cleavage, mostly downstream of the YTHDF2-binding site in the mRNA^36^. The two resulting products, 5′ and 3′ fragments, are degraded by 3′–5′ exoribonucleolytic cleavage and 5′–3′ exoribonucleolytic cleavage, respectively^39–43^.

It should be noted that both HRSP12 and CNOT1 bind to the unstructured N-terminal region of YTHDF2, but at different residues^33,36^. Amino acids 101‒200 in the N-terminal half of YTHDF2 are required for binding to CNOT1^33^. In contrast, HRSP12 efficiently interacts with amino acids 1‒100 as well as a truncated N-terminal YTHDF2 lacking amino acids 101‒200^36^. Therefore, the discrete binding residues in YTHDF2 can activate two distinct pathways for the decay of m^6^A-containing mRNA, either deadenylation by the YTHDF2–CCR4–NOT complex or endoribonucleolytic cleavage by the YTHDF2–HRSP12–RNase P/MRP complex, depending on whether HRSP12-binding sites are present in the m^6^A-containing mRNA. Currently, it is unknown whether components in these two pathways communicate with each other to regulate the destabilization of m^6^A-containing mRNA, and which cellular environments are responsible for the preferential activation of each decay pathway.

Recent studies have shown that, in addition to YTHDF2, other YTH domain-containing proteins are also engaged in mRNA degradation. For instance, YTHDF1, YTHDF2, and YTHDF3 share a subset of target transcripts that they destabilize^50–52^. In addition, the N-terminal half of YTHDC2, another YTH domain-containing protein, interacts with XRN1, a cytoplasmic 5′–3′ exoribonuclease^53^, suggesting that YTHDC2 recruits XRN1 and triggers rapid degradation of m^6^A-containing mRNA.

m^6^A-modified mRNA can also be targeted toward an opposite fate, depending on the m^6^A reader proteins and other RBPs (Fig. 2). For instance, a recently identified m^6^A reader protein, insulin-like growth factor 2 mRBP (IGF2BP), binds to the UGGAC motif^54^, which overlaps with the m^6^A motif, and increases the half-life of m^6^A-containing mRNA^20,25,54^. In addition to IGF2BP, other m^6^A reader proteins or RBPs, such as fragile X mental retardation protein (FMRP), Ras-GTPase-activating protein SH3 domain-binding protein (G3BP1), proline-rich coiled-coil 2 A (PRRC2A), and human antigen R (HuR; also known as ELAVL1), have been shown to stabilize m^6^A-containing mRNA at the transcriptome or gene-specific level^54–60^.

### *N*^*6*^,2′-*O*-dimethyladenosine

The first transcribed nucleotide next to the 5′ m^7^G cap structure in mRNA is generally methylated on the ribose ring at the 2′-OH position^3–7^. In particular, when the first nucleotide is adenosine, the methylated adenosine at the 2′-OH position, known as 2′-*O*-methyladenosine (Am), is further methylated at the N-6 position of Am, generating m^6^Am (Fig. 1)^3–7^. Therefore, m^6^Am and m^6^A are generated by very similar chemical reactions: methylation at the N-6 position of adenosine, and Am generates m^6^A and m^6^Am, respectively. However, m^6^Am has several properties that distinguish it from m^6^A. First, m^6^Am is generated by the methylation of Am, which is primarily located in the first nucleotide position adjacent to the m^7^G cap structure of mRNA^3–7^. Second, a unique writer protein, phosphorylated CTD-interacting factor 1, is responsible for generating the m^6^Am modification^61–64^, whereas a methyltransferase complex comprising METTL3, METTL14, WTAP, and KIAA1429 is involved in the generation of the m^6^A modification^22–25^. Finally, whereas m^6^A is largely demethylated by ALKBH5 but also by FTO, with a weak preference^29–32^, m^6^Am is preferentially and specifically demethylated by FTO^29,31,62^.

Although controversial^63,64^, findings from recent studies have revealed that m^6^Am-initiated mRNAs are in greater abundance and have longer half-lives than mRNAs with Am, Gm, Cm, or Um^31,62^. In vitro decapping experiments have shown that m^6^Am-initiated mRNAs are more resistant to decapping by decapping mRNA 2 (DCP2)^65^, resulting in the increased mRNA stability (Fig. 2)^31^. Furthermore, m^6^Am-initiated mRNAs are more resistant to microRNA-mediated mRNA degradation^31^, which also involves decapping (Fig. 2)^66^. Further investigation is needed to understand the molecular mechanism underlying the stabilization of m^6^Am-initiated mRNAs.

### 8-Oxo-7,8-dihydroguanosine

The bases in RNA are vulnerable to various forms of chemical damage, such as those induced by reactive oxygen species (ROS), ultraviolet light, and alkylating agents^11^. In particular, ROS—including superoxide, hydroxyl radicals, and hydrogen peroxide—are produced as byproducts of normal oxygen metabolism (e.g., cellular respiration in the mitochondria) and are also generated by various environmental stresses, such as ultraviolet irradiation and heat shock^67^. It should be noted that ROS oxidize RNA bases and generate numerous forms of oxidized RNAs^11,67^. These oxidized bases include 8-oxoG, 8-oxo-7,8-dihydroadenosine, 5-hydroxyuridine, 5-hydroxycytidine, and cytosine glycol (Fig. 1). Among these forms, 8-oxoG (an oxidized form of the guanine base) is the most abundant within mammalian cells, and its accumulation is associated with many neurodegenerative diseases^68,69^.

The oxidation of mRNA affects multiple steps of mRNA fate determination, including mRNA stability and translation^12,70–73^. For instance, the oxidation of mRNA (typically 8-oxoG) inhibits the efficiency of peptide bond formation by >1000-fold, regardless of the codon position^71^. This inhibition at the elongation step of translation causes the accumulation of stalled ribosomes^71^, which triggers the rapid mRNA degradation via the no-go decay (NGD) pathway, one of the mRNA surveillance pathways in eukaryotes (Fig. 2)^41,74–76^. The NGD pathway identifies stalled ribosomes caused by various impediments to translation elongation, such as robust secondary structures or stretches of rare codons in the mRNA^77^. The stalled ribosome is then disassembled from the mRNA and recycled. Concomitantly, the mRNA is rapidly degraded by the NGD pathway, with the coordinated action of HBS1 and DOM34^77^. Recently, CUE2 was identified as an endoribonuclease that initiates the internal cleavage of NGD targets upstream of the stalled ribosome^78^. The resulting 5′ and 3′ fragments generated by endoribonucleolytic cleavage are degraded via the exosome complex and XRN1, respectively. In this way, NGD minimizes the production of truncated polypeptides (which are potentially detrimental to cells) from the ribosomes stalled because of the mRNA oxidation.

In addition to NGD, 8-oxoG–containing mRNAs are degraded by 8-oxoG reader proteins via unknown mechanisms (Fig. 2). Y-box binding protein 1 (YBX1) and AU-rich element RBP 1 (AUF1; also known as hnRNP D) have been shown to preferentially bind to 8-oxoG, thereby triggering the rapid degradation of 8-oxoG–containing mRNAs^79,80^. A recent study also identified poly(rC)-binding protein 1 (PCBP1) as an 8-oxoG reader protein. Notably, unlike AUF1, which recognizes a single 8-oxoG, the binding of PCBP1 to RNA requires two 8-oxoGs located in close proximity. In addition, the binding of PCBP1 to oxidized RNA is associated with apoptosis—under conditions of oxidative stress^81^—rather than with the rapid degradation of 8-oxoG–containing mRNAs, as has been observed in the case of AUF1^80^. Several important questions regarding the molecular mechanisms of mRNA decay remain unanswered. First, how do 8-oxoG reader proteins trigger the rapid mRNA degradation? Second, how do the 8-oxoG reader proteins recruit general RNA-degrading enzymes? Third, is 8-oxoG reversibly converted into a normal guanosine base by a specific enzyme, as observed in the case of many other RNA modifications?

### Pseudouridine

Ψ is generated by the C–C glycosidic isomerization of a uridine base (Fig. 1). Although Ψ was first discovered in rRNA, tRNA, and small nuclear RNAs, evidence from recent transcriptome-wide analysis of Ψ profiles in humans and yeast revealed that hundreds of human and yeast mRNAs contain Ψ^82,83^. More recently, another transcriptome-wide profiling study identified thousands of Ψ sites in human mRNAs^84^. The conversion of uridine into Ψ is catalyzed by either RNA-independent or RNA-dependent mechanisms^85,86^. In the RNA-independent mechanism, Ψ is deposited by various Ψ synthases (PUSs) with different substrate specificities, different chemical reactions, and different subcellular localizations. In contrast, the RNA-dependent mechanism is guided and catalyzed by Box H/ACA small nucleolar RNAs (snoRNAs).

The chemical properties of Ψ differ from those of uridine^85,86^. For instance, Ψ makes the phosphodiester backbone more rigid, and the base pairing between Ψ and adenine is stronger than that between uridine and adenine. Because of these properties, the presence of Ψ in mRNAs can affect the local secondary structures and the protein-coding potential of the mRNA. Therefore, despite the lack of sufficient experimental evidence, it is plausible that Ψ may directly or indirectly influence pre-mRNA splicing, mRNA translation, mRNA localization, and/or mRNA stability. Indeed, the artificially targeted conversion of U-to-Ψ in translation termination codons (UAA, UGA, and UAG) turns them into missense codons^87^. In particular, the U-to-Ψ change at premature termination codons (PTCs) can inhibit the rapid mRNA degradation triggered by nonsense-mediated mRNA decay (NMD)^87^, an mRNA surveillance mechanism by which faulty (e.g., PTC containing) mRNAs are specifically recognized and removed before the production of truncated (and potentially toxic) polypeptides (Fig. 2)^41–43^.

Several lines of evidence support the hypothesis that Ψ affects mRNA stability (Fig. 2). PUS7 deletion in yeast causes a reduction in the amount of Ψ-containing mRNAs^83^, suggesting that Ψ stabilizes mRNA. In agreement with this finding, in vitro-synthesized Ψ-containing mRNAs are more stable than unmodified mRNAs with identical nucleotide sequences in mammalian cells^88^. In contrast, another study showed that, in the eukaryotic parasite *Toxoplasma gondii*, the half-life of the mRNAs pseudouridylated by PUS1 is significantly increased in the PUS1 mutant^89^, suggesting that Ψ destabilizes mRNA. Therefore, future studies should focus on elucidating the molecular mechanism underlying Ψ-mediated regulation of mRNA stability. In addition, it should be determined whether certain RBPs have the ability to directly recognize Ψ, thereby affecting the stability of Ψ-containing mRNAs, as observed in the case of other mRNA modifications.

### 5-Methylcytidine

m^5^C is generated in transcripts by NOP2/Sun RNA methyltransferase 2 (NSUN2), which catalyzes the deposition of a methyl group at the 5 position of cytosine (Fig. 1)^90–92^. m^5^C is recognized by m^5^C reader proteins, such as ALYREF^93^ or YBX1^94,95^. It remains unknown whether m^5^C is a reversible process because m^5^C erasers have not yet been identified. Similar to other RNA modifications, this modification is also present in mRNAs^96^. Bisulfite-sequencing analysis used to determine the m^5^C landscape in the human transcriptome has revealed that m^5^C sites are highly enriched in the 3′-UTR of mRNAs or near the translation initiation codon^93,97–99^.

A possible role of m^5^C in the regulation of mRNA stability has been previously implied (Fig. 2)^98,100^. Downregulation of NSUN2 causes a decrease in the amount and the half-life of *p16*^*INK4*^ mRNA, suggesting that NSUN2 functions as a stabilizer of *p16*^*INK4*^ mRNA^100^. Furthermore, two recent studies have shown that YBX1 preferentially binds to m^5^C-containing RNA through a π–π interaction between the target RNA and two tryptophan residues (Trp45 and Trp65) in the cold-shock domain of YBX1^94,95^. This interaction contributes to the stabilization of m^5^C-containing RNA, consequently affecting physiological events, such as the MZT (a reprogramming process during which maternal transcripts are eliminated and embryonic identity is established) and oncogene activation in human urothelial carcinoma of the bladder (UCB)^94,95^. During early MZT in zebrafish, the interaction between m^5^C and YBX1 stabilizes a subset of maternal mRNAs by recruiting poly(A)-binding protein cytoplasmic 1 (PABPC1)^95^. Failure of this stabilization leads to early gastrulation defects in zebrafish embryos^95^. Another recent report also showed that, in human UCB, a subset of oncogenic mRNAs have hypermethylated m^5^C sites and that the levels of these mRNAs are upregulated in an NSUN2-dependent manner^94^. In addition, the levels of NSUN2 and YBX1 proteins are higher in UCB than those in normal cells^94^. Mechanistically, YBX1 binds to and stabilizes oncogenic mRNAs with hypermethylated m^5^C sites (e.g., heparin-binding growth factor mRNA, which is critical for UCB progression and pathogenesis) by recruiting HuR, thus indicating an essential oncogenic role of m^5^C in UCB^94^.

### *N*^*4*^-acetylcytidine

Recent transcriptome-wide profiling of another cytidine modification, ac^4^C, in human cells showed that ac^4^C is widely distributed within noncoding RNAs and coding RNAs, with greater abundance near the translation initiation codon in mRNA (Fig. 1)^101^. mRNAs modified by ac^4^C are known to have increased half-lives and promoted translation (Fig. 2)^101^. Knocking out *N*-acetyltransferase 10 (*NAT10*) reduces the level of ac^4^C modification on RNA, indicating that NAT10 is a primary ac^4^C writer protein (RNA cytosine acetyltransferase)^101^. In yeast, orphan box C/D snoRNAs specifically guide Kre33 (a yeast homolog of human NAT10) to ac^4^C target sites in rRNA^102^, similar to the way that Ψ is guided by the H/ACA snoRNAs^85,86^. However, it remains unknown whether human NAT10 also uses box C/D snoRNAs to generate ac^4^C on its target RNAs. To date, neither ac^4^C reader protein nor an active deacetylation process has been reported. It is also unknown whether ac^4^C modification is a reversible process. Therefore, future studies should address the molecular mechanism underlying the stabilization of ac^4^C-containing mRNAs.

## Concluding remarks

Cellular mRNA levels are determined by various quantity control pathways (such as transcription, capping, splicing, and 3′-end formation) and quality control pathways (such as NMD and NGD). All of these molecular events are mediated by diverse *cis*-acting elements (e.g., nucleotide sequences and secondary structures) and *trans*-acting factors (e.g., RBPs and noncoding RNAs). Furthermore, recent advances have been made toward understanding the roles of RNA modifications in regulating mRNA stability. Although only certain types of modifications are discussed in this review, recent studies imply that several other RNA modifications might have the ability to influence mRNA stability. For instance, 2′-*O*-methylation (Nm)—in which a methyl group is added to the 2′-OH of the ribose ring—is highly enriched in the first and second transcribed nucleotides next to the cap structure^2^. The presence of Nm is known to increase the levels of peroxidasin mRNA^103^. Furthermore, in vitro decapping experiments have shown that a capped RNA with an Nm modification is resistant to hydrolysis by the decapping exoribonuclease DXO, which specifically recognizes and removes pre-mRNAs harboring a defective cap structure^104^. Therefore, these two recent reports suggest that Nm functions as an mRNA stabilizer. As they are oxidized, RNAs can also be alkylated upon exposure to alkylating agents that are either endogenously produced during normal metabolic processes or exogenously provided in the environment^11^. Bases, riboses, and the phosphate backbone of RNA are all vulnerable to alkylation because they contain oxygen and nitrogen atoms. As a result, numerous alkylated nucleosides can be generated in RNA, possibly affecting the RNA structure and/or the protein-coding potential of mRNA. A recent study even showed that alkylated mRNA is subject to rapid degradation via NGD^12^. Finally, the presence of internal *N*^7^-methylguanosine (m^7^G) in mRNA is known to promote translation. The positive charge of internal m^7^G may affect the RNA secondary structure and thereby affect the mRNA stability^105,106^.

Future investigations should aim to determine the transcriptome profiles of all RNA modifications, extend a list of RNA modifications that affect mRNA stability, and elucidate the underlying molecular mechanisms. In addition, considering that several mRNA modifications, such as Ψ, oxidation, and alkylation, are associated with mRNA surveillance pathways, it will be interesting to investigate whether mRNA surveillance pathways (NMD, NGD, and no-stop decay)^41–43,74^ are associated with the mRNA degradation caused by other types of RNA modifications.
